# The clock gene, brain and muscle Arnt-like 1, regulates autophagy in high glucose-induced cardiomyocyte injury

**DOI:** 10.18632/oncotarget.20811

**Published:** 2017-09-11

**Authors:** Li Qiao, Bingyan Guo, Hui Zhang, Rong Yang, Liang Chang, Yaling Wang, Xin Jin, Suyun Liu, Yongjun Li

**Affiliations:** ^1^ Department of Cardiology, The Second Hospital of Hebei Medical University, Shijiazhuang, Hebei, P.R. China; ^2^ The Hebei Institute of Cardiovascular and Cerebrovascular Diseases, Shijiazhuang, Hebei, P.R. China

**Keywords:** circadian clock, Bmal1 gene, autophagy, diabetic cardiomyopathy, cardiomyocyte injury

## Abstract

High-glucose-induced cardiomyocyte injury is the major cause of diabetic cardiomyopathy, but its regulatory mechanisms are not fully understood. Here, we report that a circadian clock gene, *brain and muscle Arnt-like 1* (*Bmal1*), increases autophagy in high-glucose-induced cardiomyocyte injury. We constructed a hyperglycemia model with cultured cardiomyocytes from neonatal rats. High-glucose-induced inhibition of autophagy and cardiomyocyte injury were attenuated by *Bmal1* overexpression and aggravated by its knockdown. Furthermore, autophagy stabilization by 3-methyladenine or rapamycin partially suppressed the effects of altered *Bmal1* expression on cardiomyocyte survival. Mechanistically, *Bmal1* mediated resistance to high-glucose-induced inhibition of autophagy at least partly by inhibiting mTOR signaling activity. Collectively, our findings suggest that the clock gene *Bmal1* is a positive regulator of autophagy through the mTOR signaling pathway and protects cardiomyocytes against high-glucose toxicity.

## INTRODUCTION

Diabetic cardiomyopathy refers to a disease process that affects the myocardium in the diabetic heart, thereby causing a variety of structural abnormalities and ultimately leading to left ventricular hypertrophy and dysfunction [1, 2]. Hyperglycemia is an independent risk factor for diabetic cardiac injury, the development and progression of diabetic cardiomyopathy are closely associated with the duration and severity of high glucose [3–6]. However, current treatments cannot effectively reduce diabetic cardiomyopathy in diabetic patients, indicating that other mechanisms remain to be discovered.

Numerous studies in both human subjects and animal models have confirmed that perturbations of the internal clock system constitute risk factors for such disorders as obesity, type 2 diabetes, and cardiovascular disease [7–11]. The circadian clock is an evolutionarily conserved fundamental mechanism in organisms, a biological response to Earth's diurnal cycles. In mammals, circadian rhythms are driven by a group of clock genes that include *Bmal1*, *Clock*, *Cry*, and *Per*. These genes are rhythmically expressed in the suprachiasmatic nucleus (SCN), the master clock that resides in the hypothalamus, and in nearly all peripheral tissues, where they control the expression of various target genes in a circadian manner, thus affecting many biochemical and physiological processes [12–14]. The clocks are present even when cells are in culture, indicating that the basic mechanism is intrinsic and self-sustained [15]. As a core clock gene, *Bmal1* plays a key role in the generation and maintenance of circadian rhythms. *Bmal1*-knockout mice display a complete loss of circadian rhythms [16]. In addition, alterations of circadian clock activity are present in the Streptozotocin-induced diabetic heart [17]. Genetic disruption of *Bmal1* induces an abnormal metabolic phenotype characterized by impaired gluconeogenesis, hyperleptinemia, glucose intolerance, and dyslipidemia [18–20]. Given this evidence, we hypothesized that the circadian gene *Bmal1* might participate in the pathogenesis of diabetic cardiomyopathy and that such a connection would furnish new opportunities for mechanism-based diabetic cardiomyopathy therapeutics.

Autophagy is a dynamic process in which damaged or aged intracellular organelles and long-lived proteins are sequestered into double-membraned vesicles termed autophagosomes. Autophagosomes then fuse with lysosomes for the degradation and recycling of their contents [21–25]. It has been reported that high-glucose inhibits autophagy in cardiomyocytes, while autophagy suppression is protective against high-glucose-induced cardiomyocyte injury [26]. These results indicate an association between altered autophagy and diabetic cardiac injury.

In this study, we sought to address 3 questions. (1) Does the circadian clock gene *Bmal1* impact cardiomyocyte death and survival under high-glucose conditions? (2) How is altered autophagy involved in the impact of altered *Bmal1* expression on cardiomyocyte viability in response to high glucose? (3) What is the molecular mechanism underlying the change in autophagy activity in response to alterations in *Bmal1* expression? Our results demonstrated that the clock gene *Bmal1* regulates autophagy via the mTOR pathway and protects cardiomyocytes against high-glucose toxicity.

## RESULTS

### Effects of altered *Bmal1* expression on cardiomyocyte viability in response to high glucose

**Figure 1:**
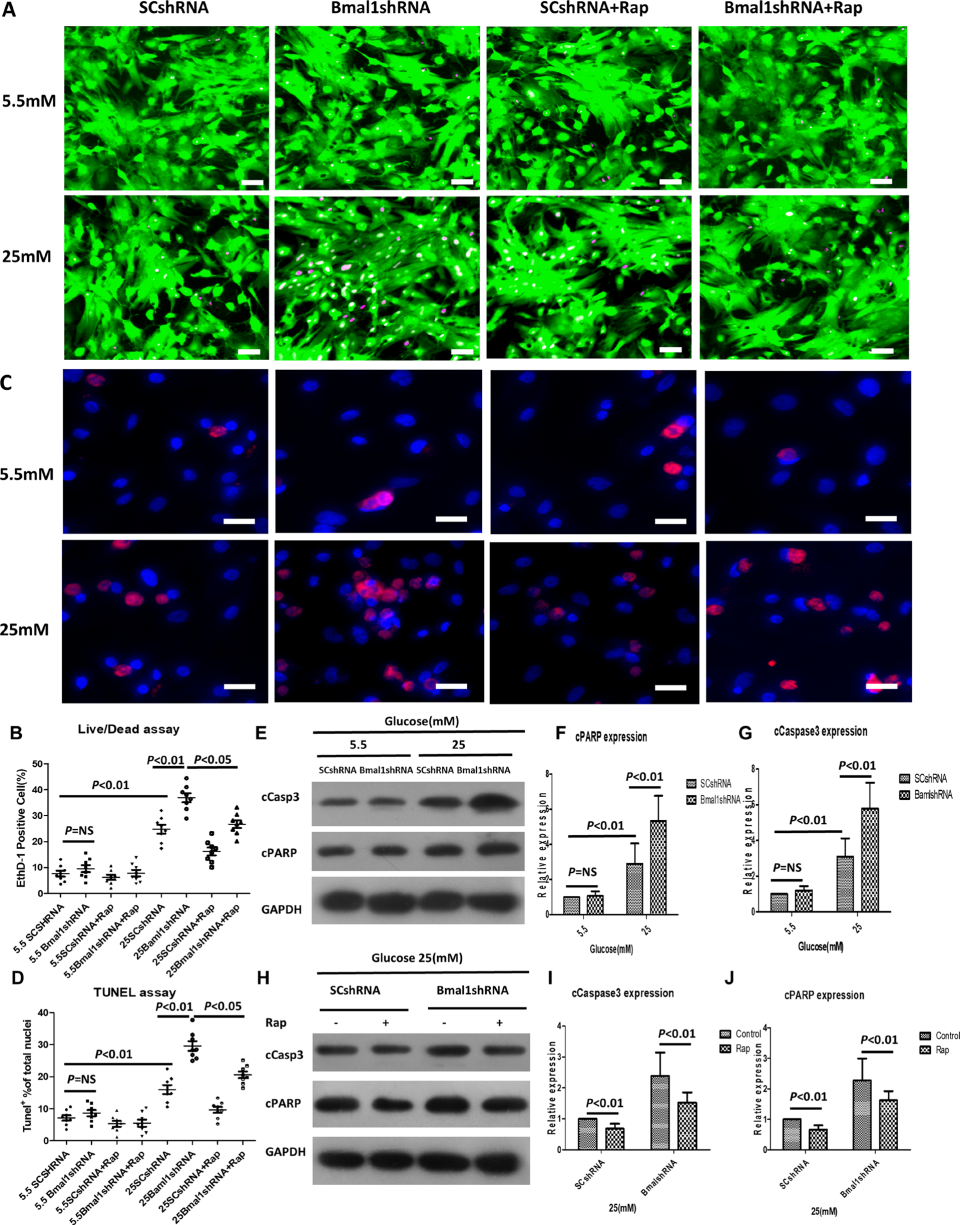
Knockdown of Bmal1 enhances high-glucose-induced cardiomyocyte death, whereas Rap treatment reverses this effect Cell death was determined by live/dead assay (**A**, **B**), apoptosis was measured by TUNEL assay (**C**, **D**), and cleavage of caspase3 (**E**, **F**, and **H**, **I**) and PARP (E, **G**, and H, **J**). (A) Representative fluorescent micrographs showing cardiomyocytes viability (Live, green fluorescent calcein-AM; dead, red fluorescent ethidium homodimer-1). (B) Quantification of dead cells by live/dead assay (*n* = 8). (C) Representative fluorescent micrographs showing apoptosis (Apoptotic cells, red; DAPI for nucleus, blue). (D) Quantification of apoptotic cells by TUNEL assay (*n* = 8). (E, H) Cleavage of caspase3 and PARP expression were analyzed by western blotting. Bands of interest were first normalized to GAPDH, and then compared with control (E, Bmal1shRNA in 5.5 mM group. H, Bmal1shRNA with no Rap), which was defined as 1. (F, I) Quantification of cleavage of caspase3 expression (*n* = 8). (G, J) Quantification of cleavage of caspase3 expression (*n* = 8). Data were expressed as the mean ± SEM, and analyzed by two-way ANOVA. *p* = NS indicates *p* > 0.05. Abbreviations: SCshRNA, cardiomyocytes were infected with SCshRNA for 18 h and then cultured under the indicated glucose conditions. Bmal1shRNA, cardiomyocytes were infected with Bmal1shRNA for 18 h and then cultured under the indicated glucose conditions. Baml1shRNA+Rap, cardiomyocytes were infected with Bmal1shRNA for 18 h and then cultured under the indicated glucose conditions for 24 h, and treated with Rap (100 nM) for another 24 h. Rap, autophagy inductive agent rapamycin. EthD-1, ethidium homodimer-1. cCasp3, cCaspase3.

Disruption of the core clock gene *Bmal1* leads to the complete loss of circadian rhythms under free-run conditions [16]. We, therefore, used lentivirus to deliver a short hairpin RNA (shRNA) against *Bmal1* mRNA that would knock down (KD) the expression of the *Bmal1* gene in cultured neonatal rat cardiomyocytes (NRCMs, Supplementary Figure 1A and 1B). We then exposed the cells to normal (5.5 mM) and high (25 mM) concentrations of glucose to study the effects of circadian disruption on cardiomyocyte survival in response to high glucose. Supplementary Figure 2 shows alteration of Bmal1 expression in transduced cardiomyocytes. Interestingly, we found that the effects of altered *Bmal1* expression on cardiomyocyte viability were detectable only when cells were exposed to high glucose. Compared with the scrambled control (SC) shRNA group, KD of *Bmal1* led to a significant increase in cardiomyocyte death under high-glucose conditions (36.92 ± 4.88% in Bmal1shRNA vs. 24.80 ± 4.88% in SCshRNA, *p* < 0.01, *n* = 8; Figure 1A and 1B). The proportion of apoptotic cells in the *Bmal1* silencing group were dramatically higher, at 30%, compared with 16% in the SC shRNA control group (*p* < 0.01, *n* = 8; Figure 1C and 1D). The exacerbated apoptosis was confirmed by examining the abundance of cleaved caspase 3 and PARP (Figure 1E–1G).

**Figure 2:**
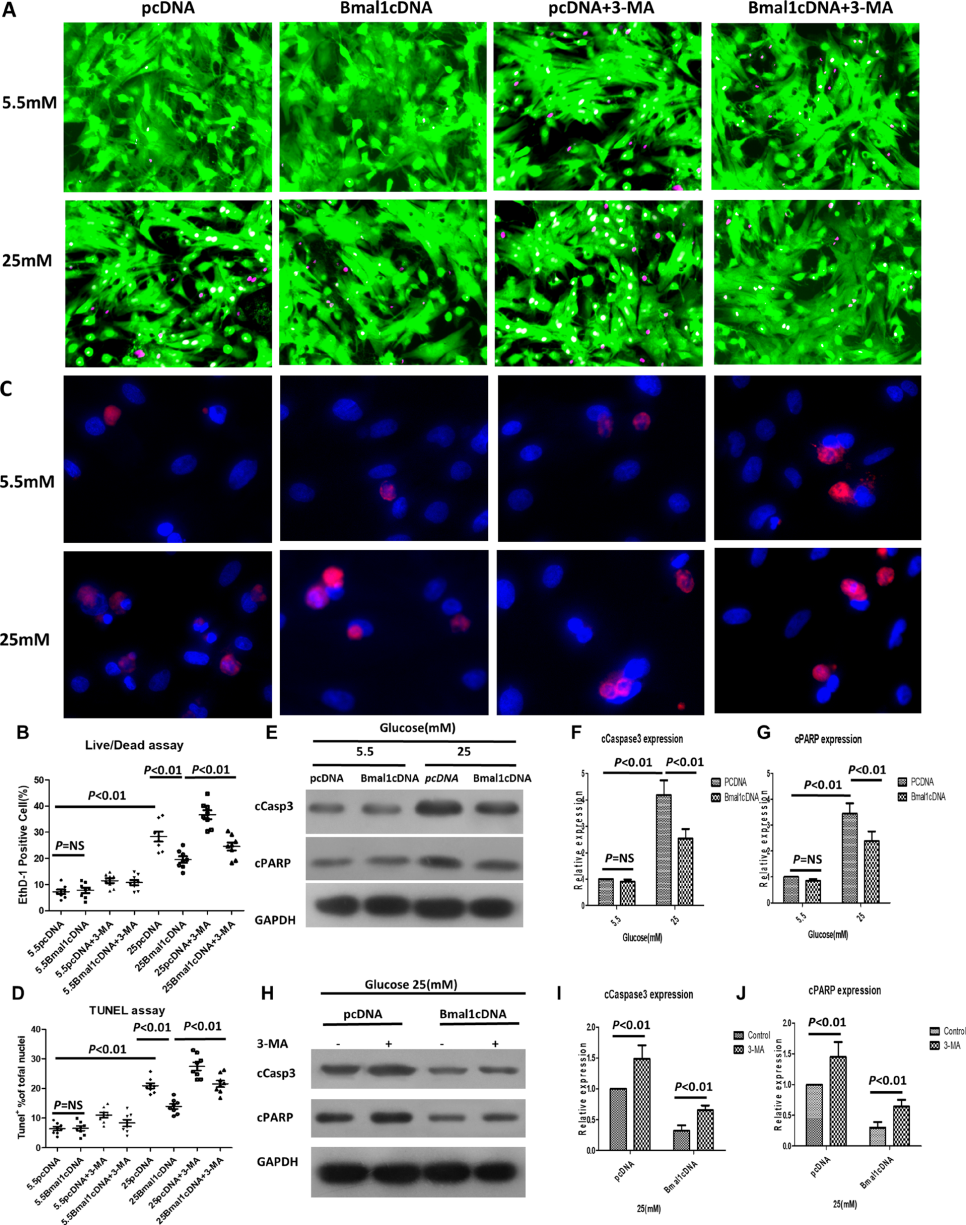
Overexpression of Bmal1 reduces high-glucose-induced cardiomyocyte death, whereas 3-MA treatment reverses this protective effect Cardiomyocyte death was determined by live/dead assay (**A** and **B**), apoptosis was measured by TUNEL assay (**C** and **D**), and cleavage of caspase3 (**E**, **F** and **H**, **I**) and PARP (E, **G** and **H**, **J**). (A) Representative fluorescent micrographs showing cardiomyocytes viability (Live, green fluorescent calcein-AM; dead, red fluorescent ethidium homodimer-1). (B) Quantification of dead cells by live/dead assay (*n* = 8). (C) Representative fluorescent micrographs showing apoptosis (Apoptotic cells, red; DAPI for nucleus, blue). (D) Quantification of apoptotic cells by TUNEL assay (*n* = 8). (E, H) Cleavage of caspase3 and PARP expression were analyzed by western blotting. Bands of interest were first normalized to GAPDH, and then compared with control (E, pcDNA in 5.5 mM group. H, pcDNA with no 3-MA), which was defined as 1. (F, I) Quantification of cleavage of caspase3 expression (*n* = 8). (G, J) Quantification of cleavage of caspase3 expression (*n* = 8). Data were expressed as the mean ± SEM, and analyzed by two-way ANOVA. *p = NS* indicates *p > 0.05*. Abbreviations: pcDNA, cardiomyocytes were infected with pcDNA for 18 h and then cultured under the indicated glucose conditions. Bmal1cDNA, cardiomyocytes were infected with Bmal1cDNA for 18 h and then cultured under the indicated glucose conditions. Baml1cDNA+3-MA, cardiomyocytes were infected with Bmal1cDNA for 18h and then cultured under the indicated glucose conditions for 24 h, and treated with 3-MA (2 mM) for another 24 h. 3-MA, autophagy inhibitor 3-methyladenine. EthD-1, ethidium homodimer-1. cCasp3, cCaspase3.

In addition, overexpression of *Bmal1* (cDNA) protected cardiomyocytes against high-glucose injury, as shown by attenuated cell death (19.54 ± 3.45% in Bmal1cDNA vs. 28.35 ± 5.46% in pcDNA, *p* < 0.01, *n* = 8; Figure 2A–2B), decreased TUNEL-positive cells (13.94 ± 2.43% in Bmal1cDNA vs. 20.90 ± 2.49% in pcDNA, *p* < 0.01, *n* = 8; Figure 2C–2D), and less cleaved caspase 3 and PARP, as shown by western blot analysis (Figure 2E–2G). However, in cardiomyocytes under normal glucose conditions, altering the expression of *Bmal1* did not obviously impact cardiomyocyte survival or death. These results revealed that alterations in *Bmal1* levels had an effect only under hyperglycemic conditions, as disruption of *Bmal1* aggravated hyperglycemic toxicity as evaluated by both cardiomyocyte death and apoptosis.

### Altered expression of *Bmal1* affects high-glucose-induced inhibition of autophagy

**Figure 3:**
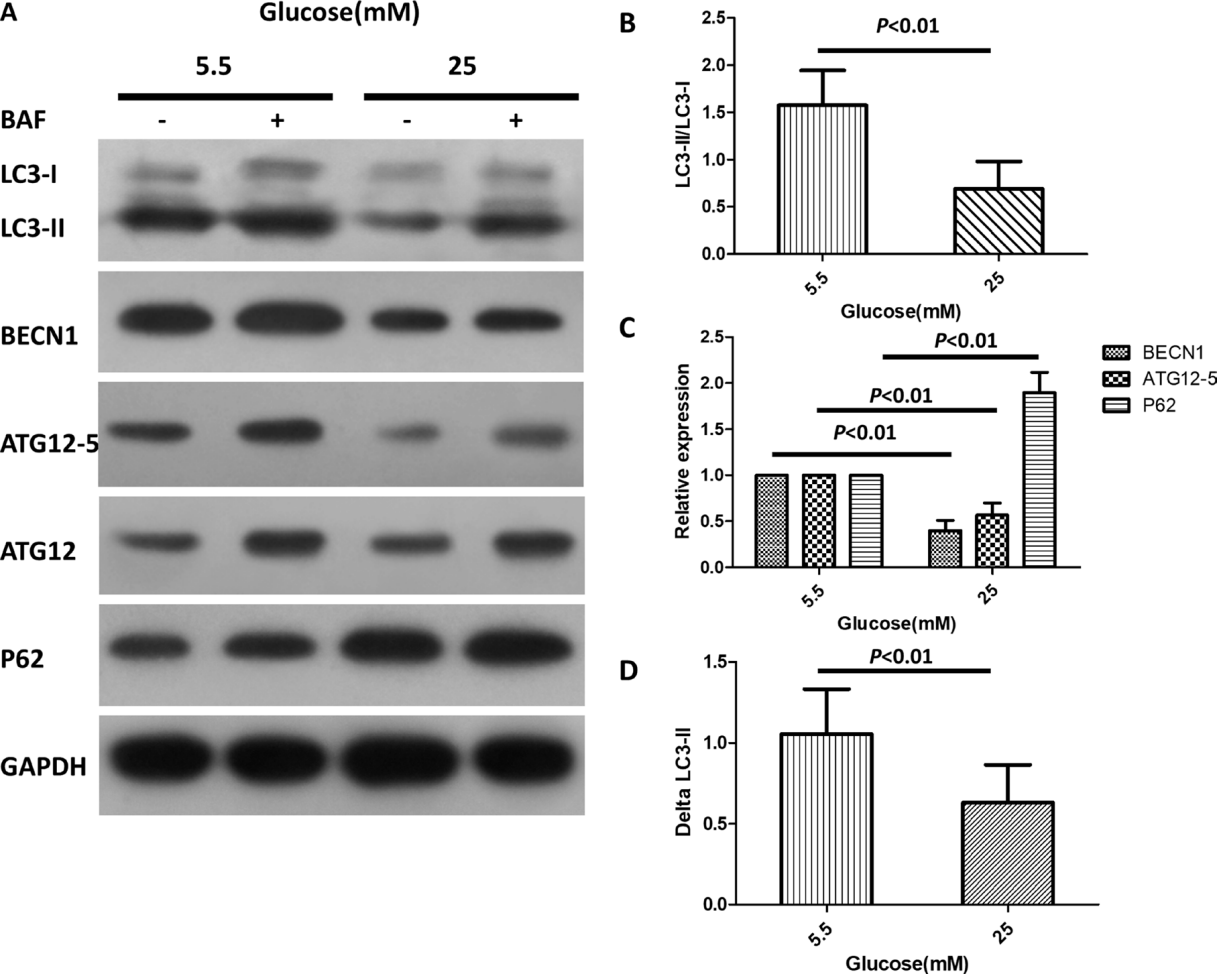
High glucose inhibits autophagy in cardiomyocytes Cardiomyocytes were cultured in DMEM with normal (5.5 mM) and high doses (25 mM) of glucose for 72 h. (**A**) Western blotting analysis showing protein levels of LC3-I, LC3-II, BECN1, ATG12–5 conjugates and p62 in the absence or presence of BAF (100 nM). (**B**–**C**) Quantification of the protein levels of LC3-II/LC3-I, BECN1, ATG12–5 conjugates and p62. The relative protein levels were first normalized with GAPDH, and then compared with control (5.5 mM glucose with no BAF) which was defined as 1. (**D**) Quantification of autophagy flux, which is defined as the difference of the LC3-II levels (ΔLC3-II) in the absence or presence of BAF. Data were expressed as the mean ± SEM, and analyzed by Student's *t-test* (B and D, *n* = 8) or two-way ANOVA (C, *n* = 8). *p < 0.05, **p < 0.01, *p = NS* indicates *p > 0.05*. Abbreviations: BAF, lysosome inhibitor bafilomycin A1. BECN1, beclin-1.

High-glucose-induced suppression of autophagy is reportedly associated with diabetic cardiac injury [26]. We therefore investigated whether *Bmal1* can influence cardiomyocyte survival in hyperglycemic conditions by mediating autophagic activity. Autophagosome is the key structure in autophagy (Supplementary Figure 1C and 1D), Beclin-1 (BECN1), microtubule-associated protein light chain 3 (LC3), ATG12-5, and p62 were used as autophagy-related markers. The levels of Beclin1, LC3-II/LC-I and ATG12–5 conjugate are proportional to the number of autophagosomes, while the level of p62 has a negative correlation with autophagic activity [27–31]. As shown in Figure 3A–3C, the levels of LC3-II/LC-I, BECN1, and ATG12–5 conjugates in cardiomyocytes exposed to high glucose were markedly lower than those in cells cultured under normal glucose concentrations, while the p62 protein levels were higher in 25 mM glucose than in 5.5 mM glucose. As a more accurate assessment of autophagy, autophagic flux reflects the number of autophagosomes that are delivered to and degraded in the lysosome and can be detected by the difference between LC3-II protein levels in the absence or presence of lysosomal inhibitors (e.g., bafilomycin A1) [32]. The autophagic flux is shown in Figure 3D (0.69 ± 0.28 in 25 mM glucose vs. 1.57 ± 0.37 in 5.5 mM glucose, *p* < 0.05, *n* = 8) as the difference in LC3-II levels (ΔLC3-II = the levels of LC3-II in the presence of BAF - those in the absence of BAF). This analysis clearly showed that high glucose inhibited autophagic activity in cardiomyocytes.

**Figure 4:**
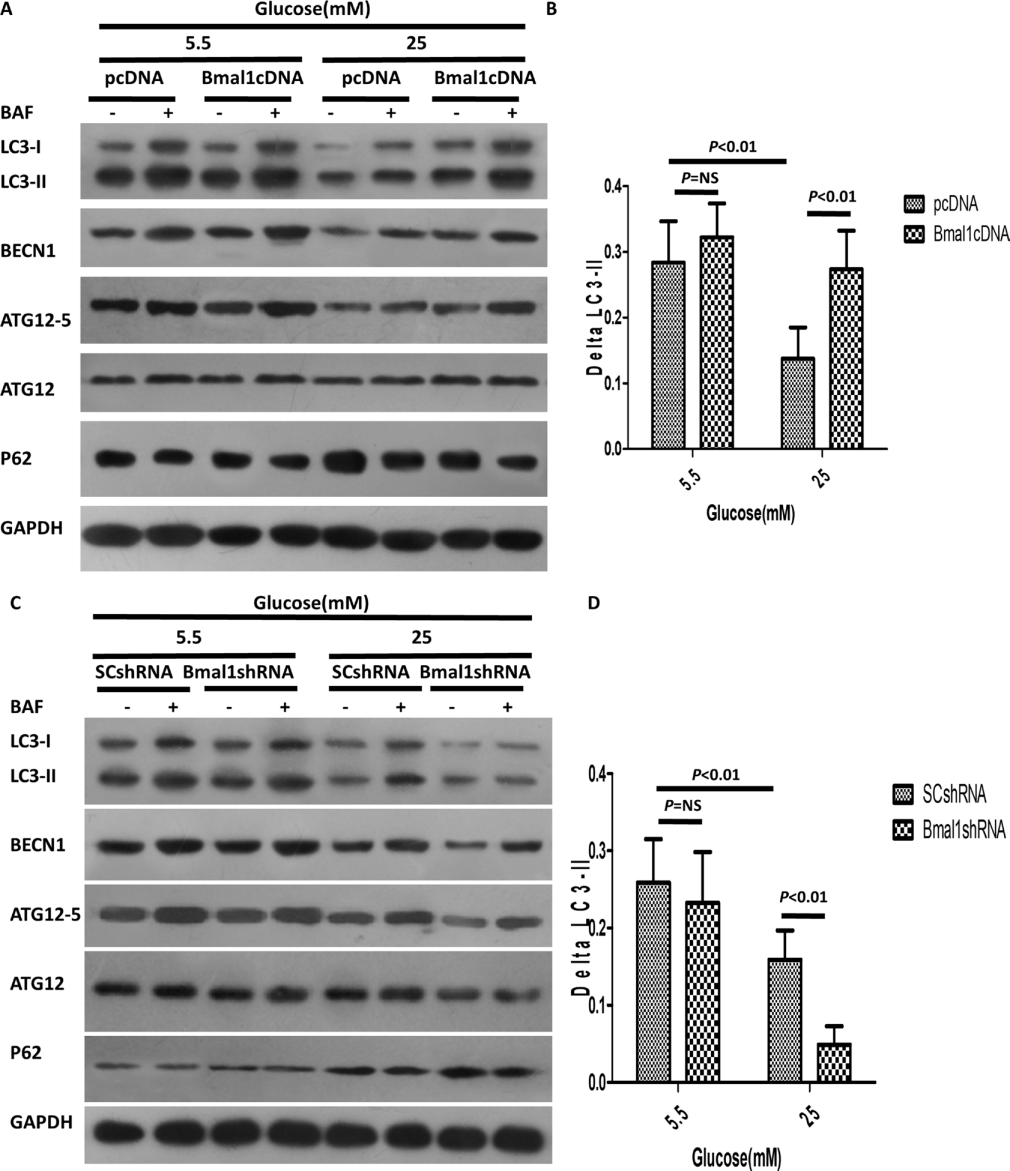
Altered Bmal1 expression affects high-glucose-induced inhibition of autophagy (**A**–**B**) High-glucose-triggered inhibition of autophagy was enhanced by overexpression of *Bmal1*. (A) Western blotting analysis showing protein levels in the absence or presence of BAF (100 nM). (B) Quantification of autophagic flux by ΔLC3-II. Overexpression of *Bmal1* increased autophagic flux only in the cardiomyocytes exposed to 25 mM glucose. (**C**–**D**) *Bmal1* KD attenuated high-glucose-induced inhibition of autophagy. (C) Western blotting analysis showing protein levels in the absence and presence of BAF (100 nM). (D) Quantification of autophagic flux by ΔLC3-II, *Bmal1* KD blocked autophagic flux only in the cardiomyocytes under high glucose condition. Cardiomyocytes were infected with lentivirus for 18 h and then cultured under the indicated glucose conditions for 48 h. pcDNA or SCshRNA was used as a control. Data were expressed as the mean ± SEM, and analyzed by two-way ANOVA (*n* = 8). *p = NS* indicates *p > 0.05*. Abbreviations: BAF, lysosome inhibitor bafilomycin A1. BECN1, beclin-1. ΔLC3-II, the difference of LC3-II levels in the absence or presence of BAF.

We further determined the effects of *Bmal1* KD or overexpression on the regulation of autophagic activity in cardiomyocytes. Interestingly, neither overexpression nor silencing of *Bmal1* had a significant effect on the autophagic activity of cardiomyocytes cultured in normal glucose concentrations. However, overexpression of *Baml1* strikingly increased the abundance of LC3-II, BECN1, and ATG12-5 in cardiomyocytes exposed to high levels of glucose (Figure 4A). Meanwhile, autophagic flux increased to 2-fold that of pcDNA cardiomyocytes, as evidenced by ΔLC3-II levels (0.27 ± 0.021 in Bmal1cDNA vs. 0.14 ± 0.017 in pcDNA, *p* < 0.01, *n* = 8; Figure 4B). These results suggested that although overexpression of *Bmal1* under hyperglycemic conditions did not further enhance autophagic flux beyond basal levels, it reversed the autophagy inhibition that was triggered by high levels of glucose. Conversely, KD of *Bmal1* further reduced autophagic flux to 30% of the SC control in cardiomyocytes cultured in 25 mM glucose (0.049 ± 0.009 in Bmal1shRNA vs. 0.159 ± 0.013 in SCshRNA, *p* < 0.01, *n* = 8; Figure 4C and 4D), indicating that high glucose and shBmal1 synergistically inhibited autophagy.

Because *Bmal1* affects both autophagy and cardiomyocyte survival under hyperglycemic conditions, we hypothesized that *Bmal1* affects cardiomyocyte survival under hyperglycemic conditions by regulating autophagic activity. If true, inhibiting autophagy would reduce the protective effect of overexpressing *Bmal1*; conversely, enhancing autophagy would reduce the injurious effects of silencing *Bmal1*. To test this hypothesis, the autophagy-inducing agent rapamycin (Rap) and the autophagy inhibitor 3-methyladenine (3-MA) were used to manipulate autophagic activity. As expected, Rap attenuated the *Bmal1*KD-induced enhancement of hyperglycemia cardiotoxicity, as shown by reduced fractions of dead cells in live/dead staining (Bmal1shRNA 36.92 ± 4.88% vs. 26.72 ± 4.10% Bmal1shRNA plus 100 nM Rap, *p* < 0.05, n = 8; Figure 1A and 1B) and TUNEL-positive cells (29.57 ± 4.30% in Bmal1shRNA vs. 20.59 ± 2.89% in Bmal1shRNA plus 100 nM Rap, *p* < 0.05, *n* = 8; Figure 1C–1D) and by lower levels of cleaved caspase 3 and PARP (Figure 1H–1J). Meanwhile, cardiomyocyte survival caused by overexpression of *Bmal1* was reduced by 3-MA, as demonstrated by increased proportions of dead cells in live/dead staining (Bmal1cDNA 19.54 ± 3.45% vs. 24.55 ± 4.28% Bmal1cDNA plus 2 mM 3-MA, *p* < 0.05, *n* = 8; Figure 2A and 2B) and TUNEL-positive cells (13.94 ± 2.42 % in Bmal1cDNA vs. 21.57 ± 3.17% in Bmal1cDNA plus 2 mM 3-MA, *p* < 0.05, *n* = 8; Figure 2C and 2D) and by enhanced cleavage of caspase 3 and PARP (Figure 2H–2J).

### The clock gene *Bmal1* induces autophagy by activating the mTORC1 signaling pathway

**Figure 5:**
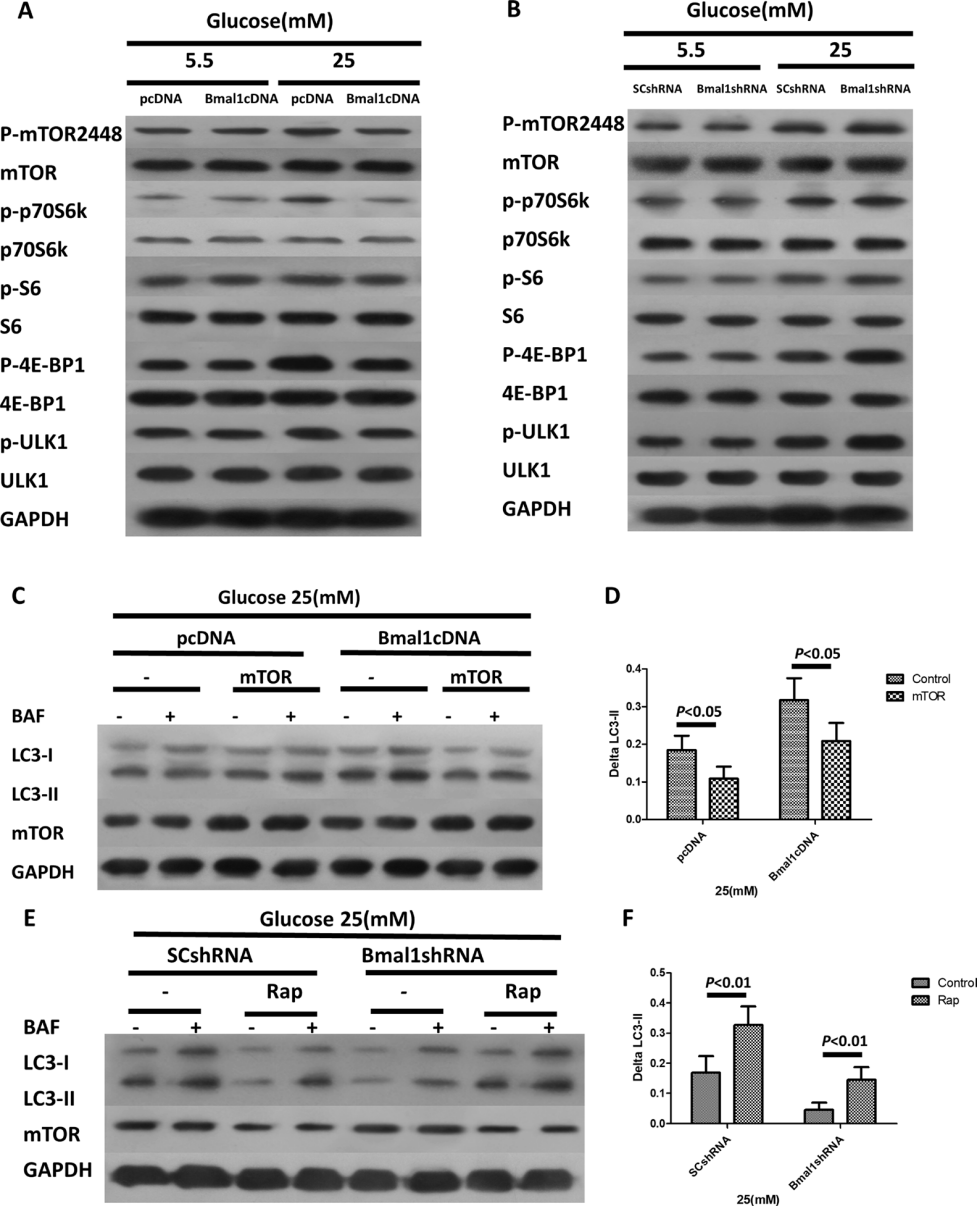
The clock gene Bmal1 induces autophagy by activation of mTORC1 signaling pathway (**A**–**B**) Western blotting analysis showing the protein expression levels and phosphorylation states of the components of mTORC1 signaling pathways in cardiomyocytes with overexpression of *Bmal1* (A) and *Bmal1* KD (B). (**C**–**D**) Rap antagonizes *Bmal1* KD –triggered suppression of autophagic flux. (D) Western blotting analysis showing protein levels in the absence or presence BAF (100 nM). (D) Quantification of autophagic flux by ΔLC3-II. (**E**–**F**) Overexpression of mTOR blocks Bmal1 overexpression- induced autophagy. (E) Western blotting analysis showing protein levels in the absence and presence BAF (100 nM). (F) Quantification of autophagic flux by ΔLC3-II. Cardiomyocytes were infected with lentivirus for 18 h and then cultured under the indicated glucose conditions for 24 h, and then treated with Rap (100 nM) or infected with mTOR for another 18 h. Data were expressed as the mean ± SEM, and analyzed by two-way ANOVA (*n* = 8). *p = NS* indicates *p > 0.05*. Abbreviations: BAF, lysosome inhibitor bafilomycin A1. BECN1, beclin-1. ΔLC3-II, the difference of LC3-II levels in the absence or presence of BAF.

The mTOR complex 1 (mTORC1) is well known to negatively regulate autophagy [33–35], and the Rap autophagy inducer that we used is an mTOR inhibitor, raising the possibility that the mTOR signaling pathway may be involved in the *Bmal1*-induced autophagic activity. We thus tested the effects of *Bmal1* KD or overexpression on mTORC1 pathway activity. Overexpression of *Bmal1* severely inhibited the mTORC1 signaling pathway, as shown by the attenuated phosphorylation of such mTORC1 downstream effectors as p70 ribosomal S6 subunit kinase (p70S6K), ribosomal S6 protein (S6), and elongation factor 4E binding protein (4EBP) (Figure 5A). Conversely, mTORC1 signaling was greatly enhanced by *Bmal1* KD (Figure 5B).

To further verify the role of mTORC1 in the regulation of autophagic activity in response to altered *Bmal1* expression, *Bmal1*-overexpressing cardiomyocytes were infected with a lentivirus expressing mTOR and then exposed to high glucose. As predicted, overexpression of mTOR decreased autophagic flux (Bmal1cDNA 0.32 ± 0.02 vs. Bmal1cDNA plus mTOR 0.21 ± 0.02, *p* < 0.05, *n*= 8; Figure 5C and 5D). Indeed, the mTORC1 inhibitor Rap antagonized the downregulation of autophagic activity triggered by *Bmal1* KD, as indicated by ΔLC3-II levels (Bmal1shRNA 0.05 ± 0.01 vs. Bmal1shRNA plus Rap 0.16 ± 0.02, *p* < 0.01, *n* = 8; Figure 5E and 5F). Together, these results demonstrated that the autophagy induction triggered by *Bmal1* is mediated by inhibition of mTORC1.

## DISCUSSION

Several molecular mechanisms have been proposed to contribute to the development of diabetic cardiomyopathy, including hyperglycemia, hyperinsulinemia, and increased circulating fatty acids and triacylglycerols, which alter various molecular pathways within the cardiomyocyte, promote cell injury, and damage cardiac function [36–38]. The failure of current treatments to reduce cardiomyopathy in diabetic patients highlights the need to identify other mechanisms that may contribute to diabetic cardiomyopathy.

It has been demonstrated that the autophagic responses to diabetic cardiomyopathy are distinctly different in type 1 and type 2 Diabetes mellitus(DM), whereas cardiac autophagic activity is enhanced in type 1 DM, it is suppressed in type 2 DM. This dichotomy of autophagy in the diabetic hearts likely reflect a net effect of insulin deficiency or resistance, varying degrees of hyperglycemia, and other diabetes-induced abnormalities such as dyslipidemia [37]. The complicated metabolic and pathological changes in the integral level may also complicate the regulation of Bmal1 to cardiomyocyte autophagy and injury. That was the reason why we decided to use the cultured cell to specifically focus on the Bmal1 gene and high glucose induced cardiomyocyte injury. Recent evidence has confirmed that alterations to circadian clock activity are present in the diabetic heart, and circadian disruption may increase susceptibility to multiple disorders, including obesity, diabetes mellitus, cardiovascular disease, and inflammation [7–11]. In this study, we demonstrated that the core clock gene *Bmal1* attenuates high-glucose-induced cardiomyocyte injury by inducing autophagic activity. This conclusion is supported by the result that overexpression of *Bmal1* markedly reversed cardiomyocyte damage and autophagy inhibition in response to high levels of glucose. Further suppression of autophagy by 3-MA attenuated the protective effect of *Bmal1* expression on cardiomyocytes. Conversely, high-glucose-triggered cardiomyocyte death and suppression of autophagic flux was further aggravated by *Bmal1* KD. Moreover, Rap-induced upregulation of autophagy reduced high-glucose toxicity. However, neither overexpression nor downregulated expression of *Bmal1* had an obvious effect on cardiomyocyte autophagy or viability at normal glucose concentrations. These results revealed that disruption of the clock gene *Bmal1* may be one of the underlying mechanisms contributing to diabetic cardiomyopathy, and this finding may provide a potential therapeutic target for reducing hyperglycemia cardiotoxicity.

Autophagy is a conserved catabolic pathway that has received increasing attention due to its important role in the cytoplasmic quality control and cell homeostasis under both normal and pathological conditions [21–25]. Autophagy has long been considered a survival mechanism that contributes to maintaining energy homeostasis and viability during nutrient or energy limitation and stress conditions [34]. However, autophagy can also cause cardiomyocyte death and heart dysfunction in other situations. Our study indicates that *Bmal1* expression increases autophagic activity in cardiomyocytes under high-glucose conditions and that this enhancement of autophagy protects cardiomyocytes against high-glucose toxicity. Our findings in cultured cardiomyocytes, as well as those of Xie et al. [39], Hiromitsu et al. [37] in type 1 DM model, are consistent with the same idea. Hiromisu et al. found that autophagy compensates in an effort to maintain cardiac function in type 1 DM, improvement of autophagy using resveratrol improved cardiac function, while diminished autophagy by chloroquine aggravated both cardiac diastolic and systolic function. [37]. Another study reported that autophagosome maturation is inhibited in high-fat-diet-induced obesity mice, which results in heart dysfunction [40]. Xie et al. report blocked autophagy in hearts of STZ-treated mice and OVE26 type 1 diabetic model mice, which is restored by treatment with metformin to improve cardiac function [39]. However, diametrically opposite reports do exist. Xu et al. reported in the same mice models, proposed inhibition of autophagy as an adaptive response that restricted cardiac dysfunction in type 1 DM [41]. Fructose-induced insulin resistance elevates autophagy, which has been suggested to contribute to cardiac pathology [42]. It is difficult to reconcile those reverse results but we would suggest that the difference might be related to the autophagy assay criteria between the studies (autophagy protein markers vs. autophagy flux). There is another *in vitro* study found that high glucose inhibits autophagy in cardiomyocytes while autophagy suppression is protective against high-glucose induced cardiomyocyte injury [26], which findings are partly inconsistent with ours. The reason for the contradictory results may attribute to the different glucose concentrations in the two studies (25 mM vs.30 mM). Various glucose concentrations may cause different levels of autophagy on cardiomyocytes, therefore leading to different effects on cell viability. Moreover, autophagy is also activated by other pathological factors, including cardiac hypertrophy [43], cardiac senescence [44], cardiac remodeling [45–46] and so on. Different extents of cardiomyocyte hypertrophy resulted from distinct levels of hyperglycemia in the two studies may also play a role in the inconsistent results. Cardiac senescence and other remodeling factors may have effects as well. Further investigation is warranted regarding these issues. Together, these results emphasize the idea that autophagy can be either protective or detrimental depending on the cell type, the cellular environment, the properties and intensity of the stimulus, and the levels of autophagy. Thus, the functional roles of autophagy under different pathological conditions should be individually determined. In addition, our results suggest that autophagy might be a molecular link between the circadian clock network and a metabolic pathway: it may act to synchronize the external nutritional status of the cells with the core circadian network to optimize nutrient collection and utilization within daily cycles of fasting and feeding. In addition to micro autophagy, studies showed alterations of other types of autophagy such as mitophagy also present in DM patients. Montaigne et al. found mitophagy was significantly decreased in diabetic myocardium, chronic hyperglycemia is a major driver of mitochondrial dysfunction in the diabetic myocardium, the disturbed mitochondrial function, which is associated with decreased contractile performance in heart tissue of diabetic patients before the onset of clinical cardiomyopathy [47]. Further study need be employed on the relationship between Bmal1 gene and mitophagy.

mTORC1 is a nutrient-sensing kinase that can be stimulated by many high-nutrient signals and that then activates anabolic processes such as protein synthesis and suppresses cellular catabolic pathways such as autophagy [33–35]. Our study indicates that mTORC1 mediates the ability of *Bmal1* to induce autophagy. First, high glucose activates mTORC1, as indicated by the increased phosphorylation of the mTORC1 substrates p70S6K, S6, and 4EBP1 (Figure 5). In addition, the mTORC1 pathway was severely attenuated by *Bmal1* overexpression and induced by *Bmal1* KD, as shown in Figure 4. Finally, the mTORC1 inhibitor Rap antagonized *Bmal1* silencing-induced autophagic activity, as indicated by ΔLC3-II levels (Figure 5C), and overexpression of mTOR dramatically decreased autophagic flux. Together, these results confirmed that the mTORC1 pathway is responsible for regulating *Bmal1*-induced autophagy in cardiomyocytes under hyperglycemic conditions.

In conclusion, our study demonstrates that the clock gene *Bmal1* protects cardiomyocytes under hyperglycemic conditions by inducing autophagy through mTORC1 signaling downregulation, suggesting a potential role for the *Bmal1* gene in autophagic activity regulation and cardiomyocyte viability. Furthermore, it is conceivable that altered *Bmal1* function resulting from disrupted circadian physiology may be a new mechanism that contributes to the prevalence of diabetic cardiomyopathy and other metabolic disorders. One limitation of this study is that autophagic flux and other parameters were determined in cultured cells. Whether our conclusions hold true in living animals requires further investigation using diabetic animal models with both gain and loss of *Bmal1* function in the heart.

## MATERIALS AND METHODS

### Neonatal rat cardiomyocyte culture under normal- or high-glucose treatment

All animal work was performed in accordance with the Institutional Animal Care and Use Committee of Hebei Medical University (Shijiazhuang, China). Neonatal rat cardiomyocytes were derived from 1- to 3-day-old Sprague-Dawley rat neonates obtained from the Laboratory Animal Center of Hebei Medical University, as described previously [48]. BrdU (5′-bromo 2′-deoxyuridine; B5002; Sigma-Aldrich, Louis, MO, USA) was applied to cardiomyocytes to inhibit cardiac fibroblast and other non-myocyte growth for 48 h, and cells were then cardiomyocytes were cultured in a glucose-free Dulbecco's modified essential medium (DMEM; 11966; GIBCO, Carlsbad, CA, USA) containing 5% fetal calf serum supplemented with 5.5 or 25 mM of glucose (G7021; Sigma-Aldrich) for 48 h, then cells were synchronized with 50% v/v adult horse serum (16050-122; GIBCO) shock treatment for 2 hours to get a cyclic expression of clock genes, after which the serum-rich medium was replaced with serum-free medium [49]. The osmolarities of all media were normalized to 25 mM with mannitol (M9647; Sigma-Aldrich). Rapamycin (BML-A275; Enzo Life Sciences, Farmingdale, NY, USA) and Bafilomycin A1 (11038; Cayman Chemical, Ann Arbor, MI, USA) were dissolved in DMSO (D2650; Sigma-Aldrich), and 3-methyladenine (M9281; Sigma-Aldrich) was dissolved in DMEM. Each drug was added to the medium to the final concentration described for each experiment.

### Transmission electron microscopy (TEM) analysis

To detect the formation of autophagosomes, cardiomyocytes were fixed with 2.5% glutaraldehyde and then in osmium tetroxide. After dehydration in an ethanol gradient, samples were incubated with propylenoid, impregnated with a mixture of propylenoid/LX-112 (1:1, 21210; Ladd Research Industries, Williston, ND, USA), and embedded in LX-112. Ultrathin sections were stained with uranyl acetate and lead citrate. Sections were examined under a Hitachi H-7500 electron microscope.

### Western blot analysis

Protein extracts from cardiomyocytes were prepared as described previously [48]. Protein samples were separated on 12% Precise Protein Gels (4561041; Bio-Rad, Hercules, CA, USA) before being transferred to polyvinylidene fluoride (PVDF) membranes (S80306, EMD Millipore, Darmstadt, Germany), blocked with 5% nonfat milk, and incubated overnight at 4°C with primary antibodies. Subsequently, the membrane was incubated with an appropriate horseradish peroxidase-conjugated secondary antibody for 1 h at room temperature in 5% nonfat milk. Blots were detected with SuperSignal West Femto substrate and exposed with the Gel Doc XR System (Bio-Rad). Bands of interest were first normalized to GAPDH and then to the control group. Data are presented as relative density ratios. Antibodies were purchased from Cell Signaling Technology (Danvers, MA, USA) as follows: LC3 (2775), ATG12 (2010), Beclin1 (3738), SQSTM1/p62 (5114), cleaved caspase 3 (9661), PARP (9542), mTOR (2972), phospho-mTOR (Ser2448, 2971), ULK1 (R600, 4773), phospho-ULK1 (Ser467, 4634), 4EBP (9644), phospho-4EBP (Ser65, 9451), p70 S6 Kinase (S6K, 9202), phospho-p70 S6K (Thr389, 9205), S6 ribosomal protein (S6, 2217), phospho-S6 (Ser240/244, 2215), and glyceraldehyde-3-phosphate dehydrogenase (GAPDH; 5174).

### Construction and utilization of replication-deficient lentivirus

*Bmal1* shRNA and cDNA constructs were generated as described previously [50]. *Bmal1* shRNA constructs in the pGIPZ vector (VGM5520-99941526, VGM5520-99342254, VGM5520-99211363) and a scrambled control (SC) vector (RHS4346) were purchased from Open Biosystems (Lafayette, CO, USA) and were constructed according to the manufacturer's instructions. cDNAs (in the pcDNA vector) were kindly provided by Dr. Ke Ma [51].

### Analysis of cardiomyocyte viability

Cardiomyocyte viability was calculated with the Live/Dead Viability/Cytotoxicity Assay Kit (R37601; Thermo Fisher Scientific, Waltham, MA USA), which estimates the number of dead cells regardless of the cause of death. The numbers of live and dead cells in 4 random microscopic fields were counted based on green fluorescent calcein-AM (live) and red fluorescent ethidium homodimer-1 (dead) staining.

### Analysis of cardiomyocyte apoptosis

To detect cardiomyocyte apoptosis, a terminal deoxynucleotidyl transferase dUTP nick-end labeling (TUNEL) assay was performed, along with an analysis of caspase 3 and PARP cleavage. TUNEL assays were performed according to the manufacturer's instructions (Roche, Basel, Switzerland, USA), and cells were then treated with DAPI (LifeTech, Carlsbad, CA, USA) to stain nuclei. Caspase 3 and PARP cleavage were detected by western blotting.

### Statistical analysis

All statistical analyses were performed with GraphPad Prism (GraphPad Software, La Jolla, CA, USA). All results are presented as the means ± SEM. Comparisons between 2 groups were performed with 2-way analysis of variance (ANOVA) followed by a Bonferroni pos*t*-test, with the exception of the comparison between 5.5 mM and 25 mM glucose, where autophagic flux was examined with a 2-tailed unpaired Student's *t*-test. Differences were considered statistically significant at *p* < 0.05.

## SUPPLEMENTARY FIGURES



## Abbreviations

3-MA: 3-methyladenine
4EBP: elongation factor 4E binding protein
BrdU: 5′-bromo 2′-deoxyuridine
BAF: bafilomycin A1
BECN1: Beclin 1
bmal1: brain and muscle Arnt-like 1
DMEM: Dulbecco's modified essential medium
LC3: microtubule-associated protein 1 light chain 3
mTOR: mammalian target of rapamycin
mTORC1: mTOR complex 1
NRCMs: Neonatal rat cardiomyocytes
PARP: Poly (ADP-ribose) polymerase
Rap: rapamycin
S6K: p70 ribosomal protein S6 kinase
SC: scrambled control
shRNA: short hairpin RNA
ULK1: unc-51-like kinase 1
