# Supplementary material for: The clock gene, brain and muscle Arnt-like 1, regulates autophagy in high glucose-induced cardiomyocyte injury
Source: Oncotarget. 2017 Sep 11;8(46):80612–24. doi: 10.18632/oncotarget.20811 (PMC5655224; doi:10.18632/oncotarget.20811)
Supplement: Supplementary file 1 [file oncotarget-08-80612-s001.pdf]

The clock gene, brain and muscle Arnt-like 1, regulates autophagy in high glucose-induced cardiomyocyte injury

SUPPLEMENTARY MATERIALS

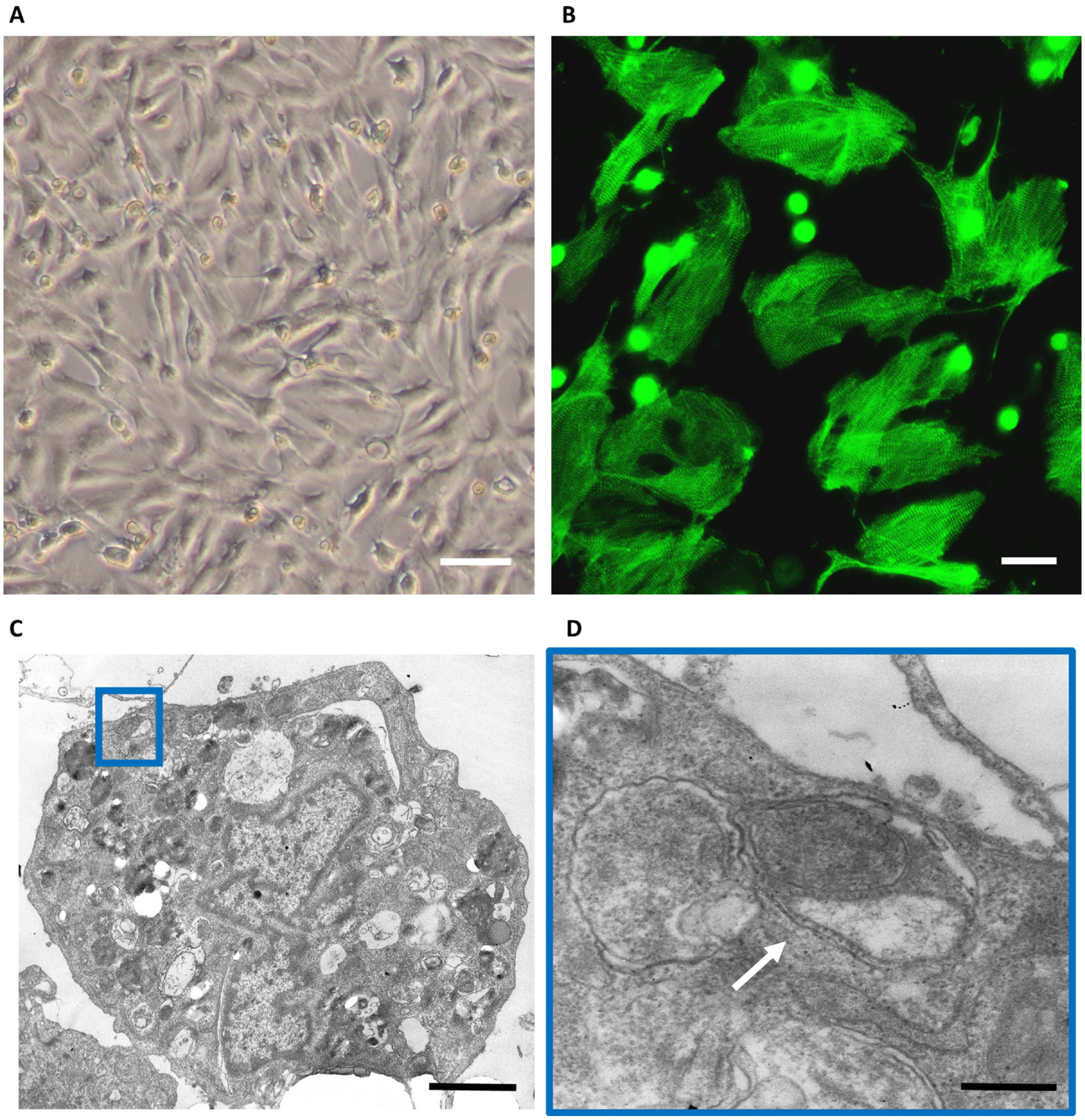

Supplementary Figure 1: Neonatal rat cardiomyocytes and transmission electron micrograph (TEM) of autophagosome in cultured cardiomyocytes. (**A**) Neonatal rat cardiomyocytes at 48 hours after primary culture. (**B**) Immunocytochemical staining for α-sarcomeric actin (α-SA) in Cardiomyocytes. (Magnification, A, ×100, scale bar = 100 um; B, ×200, scale bar = 50 um). (**C**) Cardiomyocytes were cultured in DMEM with normal (5.5 mM) glucose for 72 h, then observed by TEM. Blue box shows one autophagosome in cardiomyocyte. (**D**) High-magnification image of the blue box area in A. The autophagosome can be identified by its contents (partially cytoplasm, including mitochondrion, ribosomes and rough endoplasmic reticulum), and the electron-lucent cleft between the two limiting membranes, as a double membrane (white arrow). (magnification, A, ×4,000, scale bar = 25 um; B, ×30,000, scale bar = 300 nm).

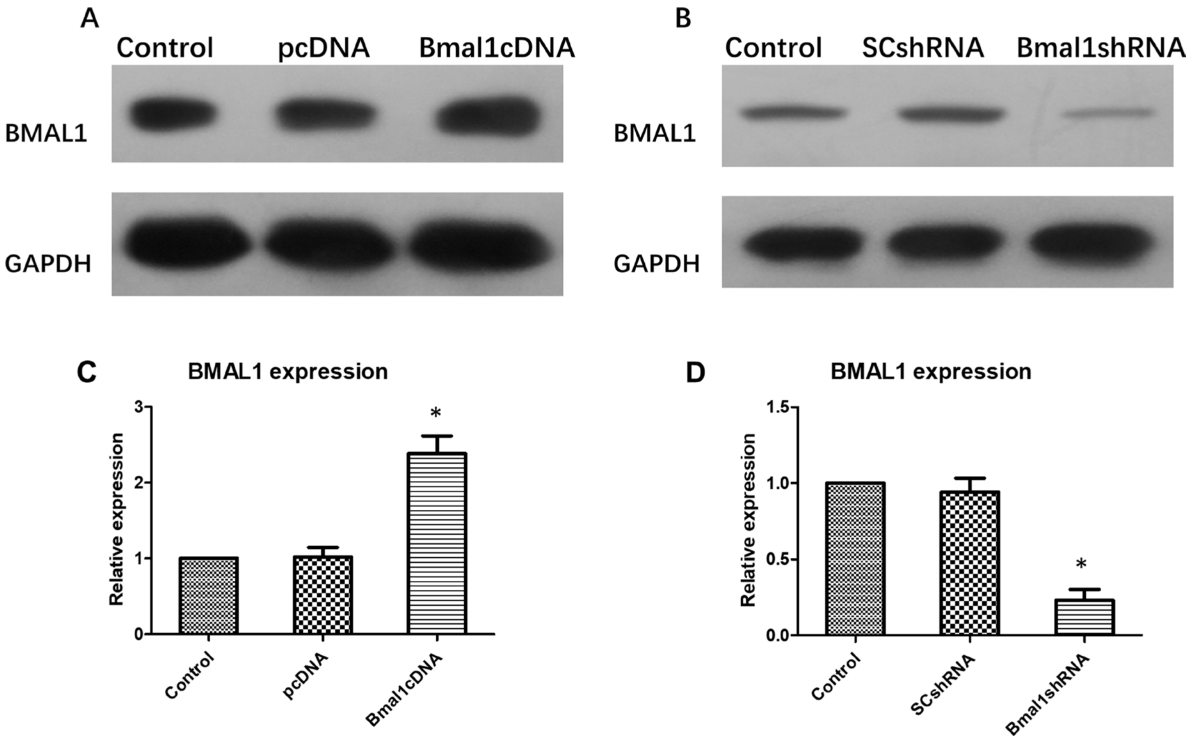

Supplementary Figure 2: Alteration of Bmal1 expression in cardiomyocytes. Cardiomyocytes were infected with Bmal1cDNA / pcDNA / Bmal1shRNA / SCshRNA for 18h, and then exposed to normal media for 72 h. (**A**, **B**) Protein levels of BMAL1 were measured by western blot analysis. (**C**–**D**) Quantification of the protein levels of BMAL1. The relative protein levels were first normalized with GAPDH, and then compared with which was defined as 1. Data were expressed as the mean ± SEM, and analyzed by one-way ANOVA (C-D, $n = 8$). *$p < 0.05$ compared with control group.
